# The effect of honey on post-tonsillectomy pain relief: a randomized clinical trial

**DOI:** 10.1016/j.bjorl.2021.08.007

**Published:** 2021-10-18

**Authors:** Azmeilia Syafitri Lubis, H.R. Yusa Herwanto, Andrina Y.M. Rambe, Delfitri Munir, Harry A. Asroel, Taufik Ashar, Aznan Lelo

**Affiliations:** a Universitas Sumatera Utara, Faculty of Medicine, Department of Otorhinolaryngology, Medan, Indonesia; b Universitas Sumatera Utara, Faculty of Public Health, Department of Environmental Health, Medan, Indonesia; c Universitas Sumatera Utara, Faculty of Medicine, Department of Pharmacology, Medan, Indonesia

**Keywords:** Tonsillectomy, Pain, Honey, Pain relief, Complementary therapy

## Abstract

**Objective:**

The study aimed to evaluate the effects of honey on the incidence of post-operative pain in patients undergoing tonsillectomy.

**Methods:**

This study is a double-blind and randomized controlled trial design. Twenty-four adult male patients underwent tonsillectomy surgery and were randomized assigned into three groups consist of honey group, placebo group, and control group. All subjects were given standard analgesia and antibiotics, also honey for the honey group and placebo for the placebo group, and only standard post-operative regimens for the control group. This study used silk-cotton tree or kapok tree honey (*Ceiba pentandra*). Honey was used by gargling every six hours for ten days. Likewise, the same method was applied in the placebo group. Pain scale was assessed for ten days using the Visual Analogue Scale questionnaire, and the frequency of analgesic drugs was recorded on days 1, 2, 4, 7, and 10.

**Result:**

Honey group showed significantly higher pain reduction when compared to placebo and control groups, with a significant reduction in the pain scale on day 1, 2, 4, 7 and 10 (*p* = 0.034; *p* = 0.003; *p* < 0.001; *p* = 0.001; *p* = 0.001) gradually; Significant differences were also observed in analgesic use, especially on day 2, 4 and 7 (*p* = 0.028; *p* = 0.001; *p* = 0.003).

**Conclusions:**

Administration of Kapok tree honey (*C. pentandra*) after tonsillectomy might reduce post-operative pain and reduce the need for analgesia. Therefore, honey can be considered a complementary medicine and can be administered routinely as adjunctive therapy for post-operative patients.

## Highlights

• Honey has effectiveness accelerates and facilitates wound healing.
• Gargling with honey led to reduced pain following tonsillectomy.
• Honey was found to lower prostaglandin levels and elevate nitric oxide.
• Honey can be used as an adjunctive regimen after surgery for better pain control.
• Honey is easy to use, safe to consume orally and available at low cost locally.

## Introduction

Tonsillectomy and adenotonsillectomy are two common surgeries in the ENT (ear, nose, and throat)· Generally, tonsillectomy is a safe procedure.^1^, ^2^ However, complications such as post-tonsillectomy pain, difficulty in swallowing, dry throat, infection, bleeding, airway obstruction, nasopharyngeal obstruction, pulmonary edema, fever, pain to jaw, otalgia, foreign body aspiration, poor healing, and vello-pharyngeal insufficiency may occur.^3^, ^4^, ^5^ Post-operative pain is the major complication during swallowing due to stimulation of tonsillar nerve endings, pharyngeal muscle spasm, and post-ingestion inflammation.^6^ Severe pain may reduce oral nutritional intake, leading to dehydration, impaired or delayed recovery after surgery.^7^ Based on some studies, antibiotics only are not effective enough to treat pain, even the addition of analgesics and steroids may not reduce pain rapidly and significantly. Another common method of controlling pain is the administration of opioids and Non-Steroidal Anti-Inflammatory Drugs (NSAIDs), which have various adverse effects.^8^ Therefore, post-operative pain control requires a method with minimum complications and maximum efficiency.^9^

One of the non-drug methods to reduce pain is honey, with various methods that have been reported in several studies. Honey has been used since ancient times to treat several ailments. Hippocrates used honey since 400 BC for healing wounds, even the ancient Egyptians used honey to treat corneal and conjunctival inflammation and burns since 5000 years ago.^10^ Honey has been shown to possess antibacterial and anti-inflammatory properties.^11^ Antioxidant, anti-inflammatory, and antibacterial properties, as well as accelerated wound recovery and pain relief, are the benefits reported for honey as a natural therapeutic method.^11^, ^12^ In modern medicine, honey has been used successfully to treat burns, graft donor sites, post-operative wound infections, skin ulcers.^13^ Moreover, honey has also been reported to benefit wound care of patients undergoing chemotherapy, those with physiological wound disorders, and prolonged injury.^13^ In previous studies, there is no report for honey side effects in wound healing.^14^ Allergy to honey is rare, but an allergic reaction to honey's proteins and allergens is possible.^13^, ^14^

Mechanical or thermal injuries may occur in the tonsillar fossa during tonsillectomy, and this location remains an open wound after surgery.^15^ Therefore, patients complain about throat pain, particularly during swallowing.^16^, ^17^ When used regularly after tonsillectomy, honey may have benefits on tissue repair, thereby reducing post-operative pains.^17^ The application of honey may reduce inflammation of infected wounds and facilitate the healing time duration.^18^

These Randomized Controlled Trials (RCTs) aimed to investigate the efficacy of honey gargle on post-tonsillectomy pain.

Level of Evidence: Systematic Review of RCT (Level I).

## Methods

This study is a randomized controlled trial. Thirty patients clinically indicated to undergo tonsillectomy and referred to ENT outpatient clinic from several hospitals, were recruited in this study. This study was approved by the Research Ethics Committee (Approval number: 61/KEP/USU/2021). Participating patients were determined based on the following inclusion and exclusion criteria (Table 1).

**Table 1:** Inclusion and exclusion criteria of study subjects.

Inclusion criteria	Exclusion criteria
Male aged 18 years old or older	Patients unwilling to participate in this study
Cooperative, well-oriented, and conscious	Cognitive deficits and limited communication skills
Sign the informed consent	Patients with diabetes mellitus, allergy to honey, or dislike to honey
The patients were diagnosed with bilateral/unilateral chronic or recurrent tonsillitis with or without obstructive symptoms	Coagulation disorders
With or without adenoidectomy	Post-operative bleeding and infection
	Postoperative-ICU admission
	Adhesions of the tonsil during surgery
	Addiction to alcohol or substances
	In a long-term treatment (e.g., chemotherapy, HIV, Tuberculosis, autoimmune diseases, etc.)

The age of subjects was 18–30 years and all subjects were male. Subjects were randomized divided into three groups with a simple random sampling technique. This study used random number tables inside the envelopes provided upon admission of the patients, stored in their folders, and could only be opened after surgery to determine their group. All study subjects underwent cold dissection tonsillectomy method, and hemostasis was controlled using monopolar diathermy. A standard post-operative regimen consisting of analgesic (mefenamic acid at a dose of 15 mg/kg, used, if necessary, a maximum dose of five times a day) and antibiotic (cefadroxil at a dose of 2 × 500 mg/day) were administrated to all post-operative study subjects, plus honey for the honey group, placebo for the placebo group, and only standard post-operative regimen for the control group.

This study used silk-cotton tree or kapok tree honey (*Ceiba pentandra*), which is certified by Indonesian Food and Drug Administration (Certificate nº DEPKES RI-137611001072). For the honey group, 15 mL honey mixed with 5 mL of water was given to the patient to gargle for two minutes, then swallowed, which is done every six hours. For the placebo group, a placebo was given to the patients (sugar syrup in honey-like concentration, consistency, and colouring), and drugs regimen with the same method as the honey group. Meanwhile, the control group was only given analgesics and antibiotics in the same way. Administration of medicine, honey and placebo was started six hours after surgery, when the patient began oral intake. The study was designed double blinded to prevent bias. None of the patients knew what their group is, also the surgeon and the researcher.

From the 1^st^, 2^nd^, 3^rd^, 7^th^, and 10^th^ day after surgery, a Visual Analogue Scale (VAS) was applied for subjective assessment of post-operative pains, while the frequency of analgesia was used for the objective evaluation. After being discharged from the hospital, all subjects were instructed to record pain and the amount of analgesia was used at home. Whenever post-operative complications such as bleeding and infection were recorded, the patient was excluded from the study.

The Statistical Package of Social Science version 24.0 (SPSS) was used to analyze data. Statistical significance was noted for a *p*-value of ≤0.05. Differences between groups in terms of VAS and the number of analgesics taken were evaluated by the Kruskal Wallis and Anova tests. Pos hoc analysis was performed using the Mann Whitney and Bonferroni tests with a significance level of 5%.

## Results

**Figure 1:**
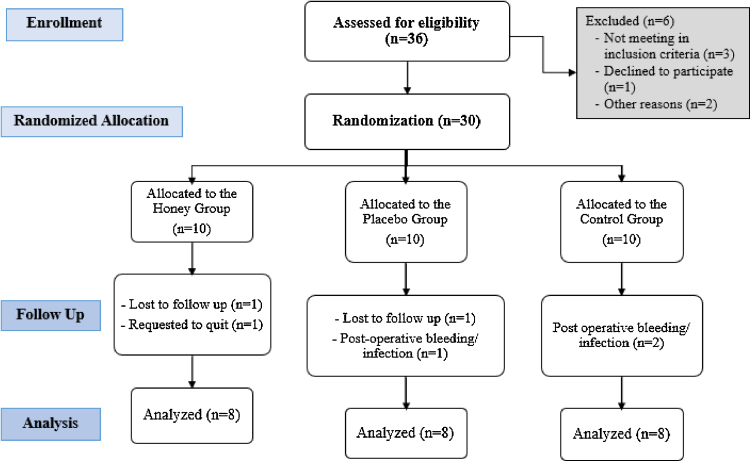
Flowchart of the trial process (CONSORT Flow Diagram).

This study covers 36 consecutive patients with the diagnosis of recurrent/chronic tonsillitis and indicated for tonsillectomy, however six patients were excluded because of not meeting in inclusion criteria, declined to participate and other reasons. A total of 30 patients underwent tonsillectomy were randomized included in the study protocol. Three patients had post-operative bleeding and infections requiring intravenous antibiotics. Furthermore, one patient requested to quit the study, and two patients were loss to follow-up. They were excluded from this study. There was a total of eight patients in each group (Honey group, Placebo group, and Control group) were finally analyzed (Fig. 1).

**Figure 2:**
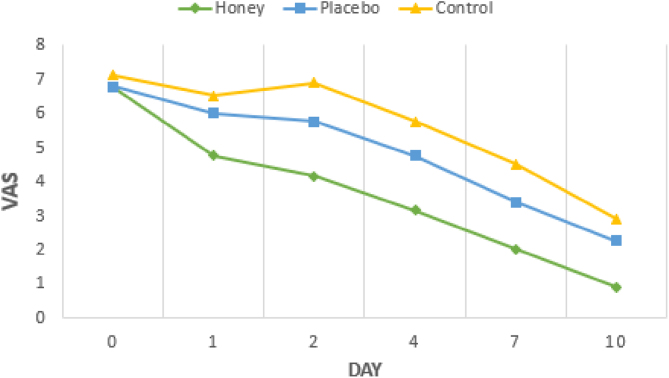
Intensity of pain scale (day 1, 2, 4, 7, and 10 after tonsillectomy).

There were significant differences of pain scale (*p* < 0.05) among the three groups on the 1^st^, 2^nd^, 4^th^, 7^th^, and 10^th^ days after surgery. The mean pain of the honey group was significantly lower than the placebo and control group. There were no significant differences of pain scale between the honey group and the placebo group on the 1^st^ day, also no significant differences of pain scale between the placebo and control groups on the 1^st^, 2^nd^, 4^th^, and 10^th^ days after surgery (Table 2 and Fig. 2).

**Table 2:** Pain scores of groups in the 1^st^, 2^nd^, 4^th^, 7^th^ and 10^th^ day after tonsillectomy (variables are expressed as mean ± SD).

Group	The intensity of pain (VAS)				
	Day 1	Day 2	Day 4	Day 7	Day 10
Honey	4.75 ± 1.28	4.13 ± 1.36	3.13 ± 0.99	2.00 ± 1.07	0.88 ± 0.64
Placebo	6.00 ± 1.07	5.75 ± 1.03	4.75 ± 0.71	3.38 ± 0.52	2.25 ± 0.25
Control	6.50 ± 1.30	6.88 ± 0.83	5.75 ± 0.89	4.50 ± 0.76	2.88 ± 0.64
**p-Value**	0.034^a^	0.003^a^	<0.001^b^	0.001^a^	0.001^a^
H vs. P	0.052^d^	0.021^d^	0.004^c^	0.012^d^	0.004^d^
H vs. C	0.020^d^	0.002^d^	<0.001^c^	0.001^d^	<0.001^d^
P vs. C	0.396^d^	0.050^d^	0.095^c^	0.010^d^	0.130^d^

H, honey; P, placebo; C, control.

a Kruskal wallis.

b Anova.

c Bonferroni.

d Mann Whitney.

**Figure 3:**
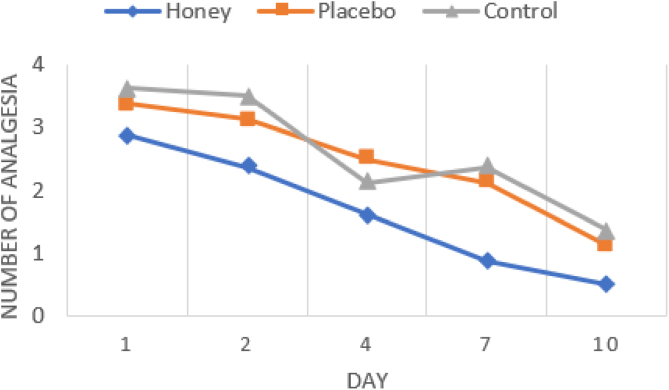
Rates of analgesia intake (day 1, 2, 4, 7, and 10 after tonsillectomy).

The number of analgesics intake per day was calculated and analyzed from day 1 to day 10. The mean analgesics intake in the honey group was significantly lower than placebo and control group, particularly at 2^nd^ day to 7^th^ day. From the 2^nd^ to 7^th^ days after surgery, the need for analgesics was significantly different (*p* < 0.05) in the honey group compared to the placebo and control groups. There were no significant differences analgesics intake between the three groups on the 1^st^ and 10^th^ day (Table 3 and Fig. 3).

**Table 3:** Number of analgesia taken after tonsillectomy (variables are expressed as mean ± SD).

Group	Number of analgesics				
	Day 1	Day 2	Day 4	Day 7	Day 10
Honey	2.88 ± 0.83	2.38 ± 0.92	1.63 ± 0.74	0.88 ± 0.64	0.50 ± 0.53
Placebo	3.38 ± 0.52	3.13 ± 0.64	2.50 ± 0.53	2.13 ± 0.64	1.13 ± 0.83
Control	3.63 ± 0.52	3.50 ± 0.53	3.25 ± 0.46	2.38 ± 0.74	1.38 ± 0.74
**p-Value**	0.122^a^	0.028^a^	0.001^a^	0.003^a^	0.069^a^
H vs. P		0.074^d^	0.025^d^	0.004^d^	
H vs C		0.015^d^	0.001^d^	0.003^d^	
P vs. C		0.232^d^	0.015^d^	0.418^d^	

^a^Kruskal Wallis; ^b^ Anova; ^c^ Bonferroni; ^d^ Mann Whitney; H, honey; P, placebo; C, control.

There is no adverse effects or allergies caused by honey were observed in the honey group.

## Discussion

Chronic tonsillitis was more commonly found in children and adolescents or young adult, and based on Sumilo’s study there was no significant difference in sex distribution.^19^

This study showed that the average pain levels of the honey group were the lowest than the placebo and the control groups. The results of this study showed a statistically significant difference (*p* < 0.05) between the honey, placebo and control groups on VAS from day 1 (*p* = 0.034) until day 10 (*p* = 0.001) postoperatively. Similar to the results of Boroundman and Lal’s study, it was found that the honey group significantly lowered the pain scale from day one.^15^, ^20^ Ozlugedik's study showed that the pain scale experienced a significant decrease in the honey group (*p* < 0.001) starting at the two first post-operative days.^10^

The difference in amount of analgesics intake was statistically significant from the 2^nd^, 4^th^, and 7^th^ day (*p* = 0.028; *p* = 0.001; *p* = 0.003). In this study, the honey group showed the lowest analgesic intake compared to the placebo and control groups. According to Ozlugedik and Boroundman, honey significantly helps reduce the frequency of analgesic intake.^10^, ^15^

In addition, in this study, the use of placebo also had an effect on pain; however, there were no significant differences in both the pain score and the frequency of analgesic intake compared to the control group. The effect of reducing the pain scale on the placebo was better on the pain scale when compared to the control group. And though there were no significant differences in both the pain scale and the frequency of analgesic use between the placebo group and the control group, the placebo group’s effect on the pain scale and the frequency of using analgesics was lower than the control group. This result may be consistent with Yaghoobi’s statement that high sugar levels have an osmotic effect and antibacterial activity on wounds, which may moisturize wounds and reduce pain.^21^ However, perhaps the placebo effect is not as good as the effect of honey on wounds.

In recent years, honey has been increasingly used in modern medicine as a “potent agent” in wound healing due to its antibacterial and anti-inflammatory effects. The mechanism for pain relief in wounds is associated with the presence of antioxidants in honey such as flavonoids, monophenols, polyphenols, vitamin C, and methylsyringate that interfere with the inflammatory amplification process by ROS (Reactive Oxygen Species).^13^, ^21^, ^22^ The clinical applications of honey, particularly in treating wounds, ulcers, and burns, are pretty striking. Honey has been reported that promotes wound epithelization, reduces inflammation, edema, and exudation, accelerates collagen synthesis, and increases the DNA content of the granulation tissue.^13^, ^23^, ^24^

After tonsillectomy, the most common morbidities are bleeding, edema, insufficient oral intake, and pain after tonsillectomy. Despite advances in anesthetic and surgical techniques, post-tonsillectomy morbidity remains a major clinical problem.^21^ Post-tonsillectomy pain is caused by mechanical and thermal injuries to the tonsillar fossa leading to post-operative inflammation, nerve irritation, and pharyngeal spasm.^10^, ^25^ With these considerations, it could be expected that honey accelerates wound recovery and reduce post-operative pain.^25^ However, it is not possible to keep honey in continuous contact with the tonsillar fossa as it is in wound dressings. Therefore, the honey application interval is done as often as possible.^15^, ^23^ In this study, honey was dissolved with water and used with direct administration by gargling then swallowed, so that the honey could reach all areas that experienced post-operative trauma, and it was performed every 6-h. Similar to Raoufian's study, subjects who received honey mixed with normal saline were gargled every 6-h for further contact of honey.^25^

This study aimed to evaluate the role of honey in controlling pain after tonsillectomy. This study used kapok tree honey (*C. pentandra*). This honey is certified and easy to obtain. Kapok tree, also known by the name of “Java cotton”, is a tropical tree that is widely grown in Asia, America, and Africa.^26^ Kapok tree honey also known has effectiveness against wounds comparable to manuka honey's properties. Kapok tree honey has been used in several clinical trials and many studies have been conducted on this honey to demonstrate its superior quality, which has potent antioxidant, anti-inflammatory, and antibacterial activity.^24^, ^27^ However, there have been many other studies examining the use of various types of honey. Therefore, any type of honey can certainly be beneficial.

No adverse effects or allergies caused by honey were observed in this study. According to Nanda, there are no side effects or resistance to honey; hence honey is considered a safe medicine.^24^ However, allergic reactions may rarely develop against some of the pollens included in honey and sometimes contain clostridia spores that lead to botulism.^16^ Therefore, the patients receiving honey should be asked whether they are allergic to honey.^17^

This study is not without limitation. First, the current study has relatively small sample size due to limited elective procedure during pandemic (COVID-19) condition. A larger sample size could possibly show significant difference outcomes. Second, VAS cannot adequately represent all aspects of pain perception because pain experienced by the patients would differ based on many factors such as personal pain threshold, psychosocial factors, length of recovery or underlying disease. Despite these limitations it remains as a widely used, validate measure of pain. Final, the minimal dose and duration that showed some beneficial effects of honey is not clearly known due to lack of data.

## Conclusion

According to our study, kapok tree honey (*C. pentandra*) used in the patient group after tonsillectomy appeared to be effective in the management of post-operative pain and may reduce the need for analgesic, without side effects or allergies. The methods used are more cost-effective, simpler, safer, and more accessible. Apart from that, honey is easy to obtain and affordable.

Therefore, this study suggests that honey as an adjuvant therapy might reduce post-operative pain and benefit in reducing post-operative analgesic requirement.

## Conflicts of interest

The authors declare no conflicts of interest.

## References

[1] Tuhanioglu B., Erkan S.O. (2018). Tonsillectomy pain control with IV dexamethasone, infiltrated dexamethasone and infiltrated bupivacaine; a randomised, double-blind, placebo controlled, prospective clinical trial. J Pak Med Assoc..

[2] Fayoux P., Wood C. (2014). Non-pharmacological treatment of post-tonsillectomy pain. Eur Ann Otorhinolaryngol Head Neck Dis..

[3] Lazim N.M., Abdullah B., Salim R. (2013). The effect of Tualang honey in enhancing post-tonsillectomy healing process: an open-labeled prospective clinical trial. Inter J Pediatr Otorhinolaryngol..

[4] Letchumanan P., Rajagopalan R., Kamaruddin M.Y. (2013). Post-tonsillectomy pain relief and epithelialization with honey. Turk J Med Sci..

[5] Hwang S.H., Song J.N., Jeong Y.M., Lee Y.J., Kang J.M. (2016). The efficacy of honey for ameliorating pain after tonsillectomy: a meta-analysis. Eur Arch Otorhinolaryngol..

[6] Cheraghi F., Almasi S., Roshanaee G., Behnud F., Tehrani T.H. (2014). Effect of parents training on controlling of pain due to tonsillectomy in hospitalized children: a randomized clinical trial study. Avicenna J Nurs Midwifery Care..

[7] Shnayder Y., Lee K., Bernstein J., Lalwani K. (2020). Current diagnosis & treatment in otolaryngology-head & neck surgery.

[8] Meo S.A., Al-Asiri S.A., Mahesar A.L., Ansari M.J. (2017). Role of honey in modern medicine. Saudi J Biol Sci..

[9] Raggio B.S., Barton B.M., Grant M.C., McCoul E.D. (2018). Intraoperative cryoanalgesia for reducing post-tonsillectomy pain: a systemic review. Ann Otol Rhinol Laryngol..

[10] Ozlugedik S., Genc S., Unal A., Elhan A.H., Tezer M., Titiz A. (2006). Can post-operative pains following tonsillectomy be relieved by honey?: A prospective, randomized, placebo-controlled preliminary study. Int J Pediatr Otorhinolaryngol..

[11] Simon A., Sofka K., Wiszniewsky G., Blaser G., Bode U., Fleishchhack G. (2006). Wound care with antibacterial honey (Medihoney) in pediatric hematology-oncology. Support Care Cancer..

[12] Shahanipour K., Sadeghi M. (2016). The therapeutic effects of aloe vera and honey on burn wounds in rats. J North Khorasan Univ Med Sci..

[13] Lazim N.M., Baharuddin A. (2017). Nutritional modulators pain aging population.

[14] Khan F., Abadin Z., Rauf N. (2007). Honey: nutritional and medicinal value. Int J Clin Pract..

[15] Boroumand P., Zamani M.M., Saeedi M., Rouhbakhshfar O., Motlagh S.R., Moghaddam F.A. (2013). Post tonsillectomy pain: can honey reduce the analgesic requirements?. Anesth Pain Med..

[16] Nanda M.S., Mittal S.P., Gupta V. (2017). Role of honey as adjuvant therapy in patients with sore throat. Natl J Physiol Pharmac Pharmacol..

[17] Al-Waili N., Salom K., Al-Ghamdi A. (2011). Honey for wound healing, ulcers, and burns; data supporting its use in clinical practice. Sci World J..

[18] Nanda M.S., Kaur M., Luthra D. (2016). Role of honey in post-operative tonsillectomy cases. Inter J Contemp Med Res..

[19] Sumilo D., Nichols L., Ryan R., Marshall T. (2019). Incidence of indications for tonsillectomy and frequency of evidence-based surgery: a 12-year retrospective cohort study of primary care electronic records. Br J Gen Pract..

[20] Lal A., Chohan K., Chohan A., Chakravarti A. (2017). Role of honey after tonsillectomy. Clin Otolaryngol..

[21] Yaghobbi R., Kazerouni A., Kazerouni O. (2016). Evidence for clinical use of honey in wound healing as an antibacterial, anti-inflammatory antioxidant and anti-viral agent: a review. Jundishapur J Nat Pharm Prod..

[22] Mohebbi S., Nia F.H., Kelantari F., Nejad S.E., Hamedi Y., Abd R. (2014). Efficacy of honey in reduction of post-tonsillectomy pain, randomized clinical trial. Int J Pediatr Otorhinolaryngol..

[23] Raofian H., Nasiri E., Ghafari R., Akbari H. (2020). The effect of gargling cold normal saline in comparison to normal saline mixed with honey on postoperative pain relief in tonsillectomy or adenotonsillectomy: a randomized clinical trial. Iran Red Crescent Medical J..

[24] Ayu P., Sundoro A., Sudjatmiko G. (2012). Antibacterial activity of indonesian local honey against strains of *P. aeruginosa*, *S. aureus* and MRSA. Jurnal Plastik Rekonstruksi (JPR)..

[25] Molan P., Rhodes T. (2015). Honey: a biologic wound dressing. Wounds..

[26] Rivers M.C., Mark J. (2017).

[27] Nayaka N.M.D., Fidrianny I., Sukrasno Hartati R., Singgih M. (2020). Antioxidant and antibacterial activities of multiflora honey extracts from the Indonesian *Apis cerana* bee. J Taibah Univ Med Sci..

